# Innate Signaling in the CNS Prevents Demyelination in a Focal EAE Model

**DOI:** 10.3389/fnins.2021.682451

**Published:** 2021-06-03

**Authors:** Magdalena Dubik, Joanna Marczynska, Marlene T. Mørch, Gill Webster, Kirstine Nolling Jensen, Agnieszka Wlodarczyk, Reza Khorooshi, Trevor Owens

**Affiliations:** ^1^Neurobiology, Department of Molecular Medicine, University of Southern Denmark, Odense, Denmark; ^2^Innate Immunotherapeutics, Auckland, New Zealand

**Keywords:** innate signaling, demyelination, focal EAE, myeloid cells, NK cells, T-cells, neuroinflammation

## Abstract

The pathological hallmark of multiple sclerosis (MS) is the formation of multifocal demyelinating lesions in the central nervous system (CNS). Stimulation of innate receptors has been shown to suppress experimental autoimmune encephalomyelitis (EAE), an MS-like disease in mice. Specifically, targeting Toll-like receptor 9 (TLR9) and NOD-like receptor 2 (NOD2) significantly reduced disease severity. In the present work we have developed a novel focal EAE model to further study the effect of innate signaling on demyelinating pathology. Focal lesions were induced by stereotactic needle insertion into the corpus callosum (CC) of mice previously immunized for EAE. This resulted in focal pathology characterized by infiltration and demyelination in the CC. We find that intrathecal delivery of MIS416, a TLR9 and NOD2 bispecific innate ligand, into the cerebrospinal fluid reduced focal lesions in the CC. This was associated with upregulation of type I and II interferons, interleukin-10, arginase-1, CCL-2 and CXCL-10. Analysis of draining cervical lymph nodes showed upregulation of type II interferons and interleukin 10. Moreover, intrathecal MIS416 altered the composition of early CNS infiltrates, increasing proportions of myeloid and NK cells and reducing T cells at the lesion site. This study contributes to an increased understanding of how innate immune responses can play a protective role, which in turn may lead to additional therapeutic strategies for the prevention and treatment of demyelinating pathologies.

## Introduction

Multiple sclerosis (MS) is a chronic inflammatory and demyelinating disease of the CNS. The pathological hallmark of MS consists of multifocal demyelinated lesions with varying degrees of inflammation and neurodegeneration (Lassmann et al., 2007; Filippi et al., 2018). There is currently no cure for MS and treatment options are dominated by anti-inflammatory and immunomodulatory agents, but these treatments do not reverse myelin loss or neurodegenerative axonal damage. Therefore, improved understanding of cellular and molecular mechanisms that prevent myelin damage is needed, to pave the way to future therapeutic strategies against demyelinating pathologies. We have previously shown that simultaneous activation of innate receptors, Toll-like receptor (TLR) 9 and Nucleotide-binding oligomerization domain (NOD) 2 within the CNS via administration of experimental microparticle MIS416 had a protective effect in experimental autoimmune encephalomyelitis (EAE), an MS-like disease in mice, that resulted in suppression of disease activity (Khorooshi et al., 2020). We have now extended these studies to evaluate the direct effect of innate signaling on demyelinating pathology by utilizing a focal EAE model. In most EAE models, CNS inflammation and demyelination are predominantly restricted to the spinal cord, and correlate with motor symptoms. Demyelinated lesions occur sporadically at unpredictable sites and with variable timing (You and Gupta, 2018), which hampers study of demyelination. Focal EAE can be induced at predictable locations by stereotactic needle insertion in immunized animals. Focal EAE models described so far have used pro-inflammatory cytokines, such as tumor necrosis factor alpha, to induce cortical lesions in rodent brains (Merkler et al., 2006; Chaudhary et al., 2015; Lassmann and Bradl, 2017). Here we have innovated a novel focal EAE model that is induced in mice, without cytokines. This approach allows for better understanding of the pathology with regards to cell infiltration, cytokine composition and general changes that occur in demyelinated lesions. The beneficial effect of innate signaling on demyelinating pathology is an aspect of disease protection that we wish to address. In the present study we used this focal EAE model to show that innate receptor signaling within the CNS can prevent the formation of demyelinated lesions.

## Materials and Methods

### Mice

Female C57BL/6J mice aged 6–8 weeks were purchased from Taconic Europe A/S and maintained in the Biomedical Laboratory, University of Southern Denmark. The experiments were conducted in accordance with the national ethical committee (Animal Experiments inspectorate under Danish Ministry of Food, Agriculture and Fisheries, The Danish Veterinary and Food Administration, approval identification number: 2020-15-0201-00652).

### Focal EAE Lesion

**FIGURE 1:**
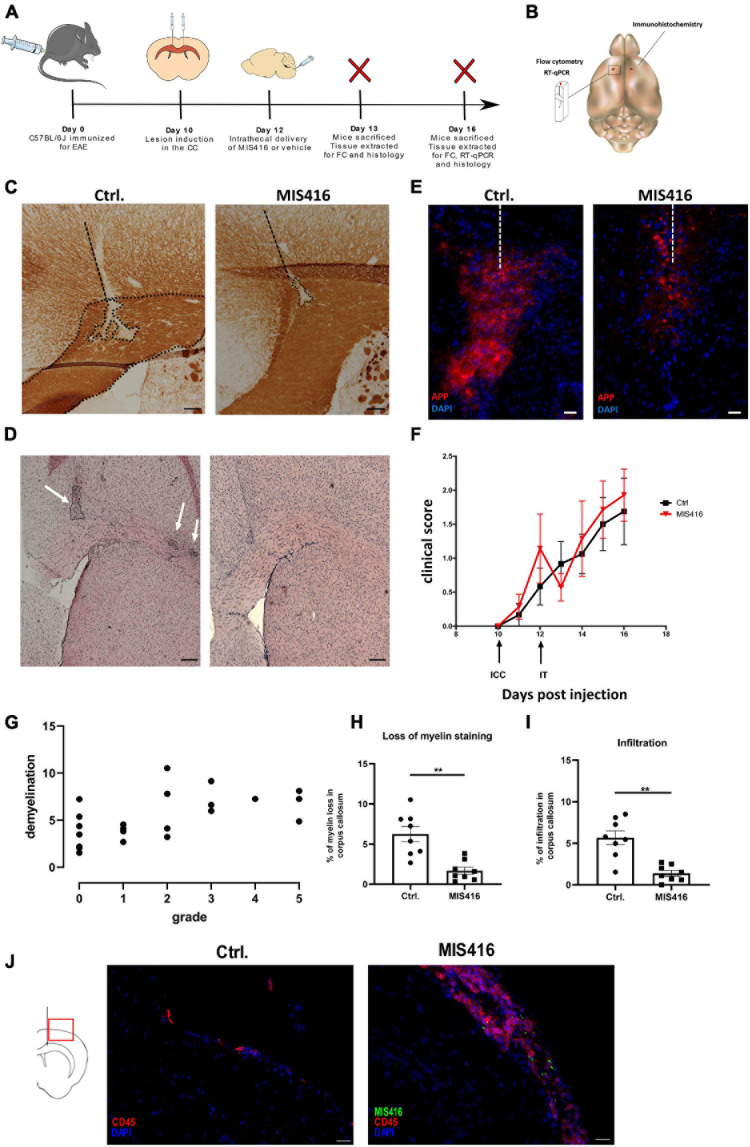
Intrathecal MIS416 reduced focal lesion pathology and altered inflammatory responses at the lesion site. **(A)** Experimental layout: C57BL/6J mice were immunized for EAE (day 0) and a focal lesion in CC was induced by a stereotactic needle insertion (day 10). Mice then received intrathecal injection with either MIS416 or vehicle (day 12). Animals were sacrificed and tissue extracted for analysis by immunohistochemistry, RT-qPCR or FC (day 13 and 16). **(B)** A Schematic illustration demonstrating the selected fragment of brain tissue surrounding the lesion site (approx. 50 mg) which was isolated for RT-qPCR and FC. The red dots indicate the injection sites. The whole right hemisphere was isolated for immunohistochemical evaluation. **(C)** Representative images of CC from vehicle (Ctrl, left panel) and MIS416-treated (right panel) groups stained with anti-MOG. Demyelination was identified by loss of MOG staining. Dotted lines represent the needle track and the area of demyelination. Scale bars: 100 μm **(D)** Representative images of CC from vehicle (Ctrl, left panel) and MIS416-treated (right panel) groups stained with H+E to show infiltration. Scale bars: 200 μm. **(E)** Immunofluorescence staining of brain section (day 16) with anti-APP (red) and nuclear staining (DAPI, blue) from mice intrathecally injected with vehicle (Ctrl) or MIS416. Scale bar: 20 μm. **(F)** Clinical scores of mice with focal EAE. Shown is pooled data from two individual experiments. MIS416 treated mice (red symbols, *n* = 8) and control mice (black symbols, *n* = 8). **(G)** Correlation between EAE grade and lesion size. The graph shows pooled data from mice with focal EAE (but otherwise untreated), from 4 different experiments (*n* = 22, *r* = 0.632, *p* = 0.0012). **(H)** Quantification of demyelination, calculated as a percentage of CC in vehicle (Ctrl, *n* = 8) and MIS416 (*n* = 8) treated groups. **(I)** Quantification of infiltration in the CC in vehicle (Ctrl, *n* = 8) and MIS416 (*n* = 8) treated groups. Data are presented as mean ± SEM. Results were analyzed using the two-tailed Mann-Whitney *u*-test; **p < 0.01. **(J)** Immunofluorescence staining of brain section (day 16) with anti-CD45 (red) and nuclear staining (DAPI, blue) from mice intrathecally injected with vehicle (Ctrl) or fluorescent MIS416 (green). Scale bar: 20 μm.

C57BL/6J mice were immunized by subcutaneous injection in the flanks with 100 μg MOGp35-55 (MOG35-55, TAG Copenhagen) emulsified in complete Freund’s adjuvant (incomplete Freund’s adjuvant (BD Difco) supplemented with 200 μg heat-inactivated Mycobacterium tuberculosis (BD Difco). At the time of immunization and the day after mice received an intraperitoneal injection of 300 ng of pertussis toxin (Sigma-Aldrich). Ten days after immunization mice were anesthetized by 2% isoflurane inhalation (Attane Vet.) and placed in a stereotactic frame (Kopf Instruments). A 30-gauge needle attached to a 50 μL Hamilton syringe was inserted sequentially into the left and right hemisphere for the formation of focal lesions in the corpus callosum (CC) (Figure 1A). Stereotactic coordinates were relative to bregma, +/−1 mm lateral, 1 mm anterior and 1.6 mm ventral. A total volume of 2 μL of vehicle (sterile phosphate buffered saline (PBS)) was infused at the rate 0.4 μL/min. Mice received buprenorphine (0.1 mg/kg of Temgesic (Fresenius Kabi)) for pain relief and 0.8 mL of isotonic sterile saline subcutaneously to hinder dehydration. Mice were monitored daily for loss of body weight and EAE symptoms. The EAE grades were as follows: Grade 0; no signs of disease; Grade 1 weak tail tonus or hook tail; Grade 2, floppy tail or complete loss of tonus in the tail; Grade 3, floppy tail and hind limb paresis; Grade 4, floppy tail and unilateral hind limb paralysis; Grade 5, floppy tail and bilateral hind limb paralysis. Due to ethical considerations, mice were sacrificed within 24 h of reaching grade 5.

### Intrathecal Injection

On day 12 after immunization (Figure 1A), mice received an intrathecal (i.t.) injection of MIS416 or vehicle directly into the cerebrospinal fluid (CSF) via cisterna magna. Mice were anesthetized using 2% isoflurane inhalation. A 30-gauge needle bent at an angle of 55°, 2 mm from the tip, and attached to a 50 μL Hamilton syringe was inserted between the skull and the cervical vertebra. A total volume of 10 μL containing either 25 μg of Alexa Fluor (AF) 488-conjugated MIS416 (White et al., 2018; Khorooshi et al., 2020) or vehicle (PBS) was injected. Following the i.t. injection, mice received 1 mL of isotonic sterile saline subcutaneously to hinder dehydration, and pain relief as previously described.

### Tissue Processing

Mice were euthanized on day 13 or 16 post immunization with an overdose of sodium pentobarbital (100 mg/kg, Glostrup Apotek) and then intracardially perfused with 20 mL ice-cold PBS. A 50 mg piece of tissue from the left-brain hemisphere that included the injection site was either transferred into tubes containing TRizol Reagent (Ambion) for RNA extraction for analysis by reverse transcriptase-quantitative polymerase chain reaction (RT-qPCR) or used for flow cytometry (FC) (Figure 1B). For histological analysis the right-brain hemisphere (Figure 1B) as well as CNS draining lymph nodes – deep cervical (dcLNs) and mandibular lymph nodes (mLNs) – were isolated. The tissues were fixed in 4% paraformaldehyde (PFA) (Sigma-Aldrich) and then immersed in 30% sucrose (Sigma-Aldrich) 4°C for 2–3 days. Tissues were then embedded in Killik cryostat embedding medium (Bio-Optica) and frozen directly afterward in 2-methylbutane (Sigma-Aldrich) using liquid nitrogen. Tissues were cut frontally on a cryostat (Leica) into 12 μm (brain) and 6 μm (LNs) thick tissue sections. Tissue from unmanipulated mice was used as additional control.

### RNA Isolation and Quantitative RT-PCR (RT-qPCR)

RNA was extracted from brain and LN tissue using TRIzol reagent in accordance with the manufacturer’s protocol. 1 μg of total RNA was converted into cDNA using M-MLV reverse transcriptase (Invitrogen) according to the manufacturer’s protocol. The following sequence-specific primers and probes were used: Interferon gamma (IFN-γ) (For: CATTGAAAGCCTAGAAAGTCTGAATAAC, Rev: TGGCTC TGCAGGATTTTCATG, probe TCACCATCCTTTTGCCAGTT CCTCCAG), Interleukin (IL)-10 (For: GGTTGCCAAGCC TTATCGGA, Rev: ACCTGCTCCACTGCCTTGCT, probe TGAGGCGCTGTCATCGATTTCTCCC), Interferon regulatory factor 7 (IRF7) (For: CACCCCCATCTTCGACTTCA, Rev: CCA AAACCCAGGTAGATGGTGTA, probe CACTTTCTTCCGAG AACT), IFN-β (Forward GCGTTCCTGCTGTGCTTCTC, Reverse TTGAAGTCCGCCCTGTAGGT, Probe CGGAAATG TCAGGAGCT MGB), IFN-α (2+6+12+14) (For: AGGATGT GACCTGCCTCAGACT, Rev: GCTGGGCATCCACCTTCTC, probe CTCTCTCCTGCCTGAAG), Monocyte chemoattractant protein-1 (CCL2) (For: TCTGGGCCTGCTGTTCACA, Rev: ACTCATTGGGATCATCTTGCT, probe CTCAGCCAGATGCA GTT), C-X-C motif chemokine 10 (CXCL-10) (For: GCC GTCATTTTCTGCCTCAT, Rev: GGCCCGTCATCGATATGG, probe GGACTCAAGGGATCC), Arginase-1 (Arg-1) (Mm0047 5988_m1, Applied Biosystems), Oligodendrocyte transcription factor 1 (Olig-1) (Mm00497537_s1), Myelin basic protein (MBP) (Mm0126640_m1), Sirtuin 2 (Sirt-2) (Mm01149204 _m1), Insulin-like growth factor 1 (Igf-1) (For: CCGAGGGG CTTTTACTTCAACAA, Rev: CGGAAGCAACACTCATCCA CAA). All samples were run as duplicates on a 7300 Real time PCR system (Applied Biosystems) and the results were normalized to 18S rRNA expression using the 2^–Δ*Ct*^ method.

### Flow Cytometry

A single cell suspension was prepared from each brain sample using a Multi Tissue Dissociation Kit #1 (Miltenyi Biotec). The dissociated tissue was forced through a 70 μm cell strainer with Hank’s buffered salt solution (HBSS, Gibco) supplemented with 2% fetal bovine serum (FBS, Merck). Myelin was then removed following centrifugation on a 37% Percoll gradient (GE Healthcare Bioscience AB). Cells were then incubated with anti-CD16/CD32 antibody (clone 2.4G2, BD Biosciences) and Syrian hamster IgG (Jackson ImmunoResearch Laboratories Inc.) to block non-specific staining and stained with fluorophore-labeled anti-CD45 (30-F11), anti-CD11b (M1/70), anti-F4/80 (BM8), anti-Gr1 (RB6-8C5), anti-TCRβ (H57-597) and anti-NK1.1 (PK136). All antibodies were purchased from BioLegend. The stained samples were evaluated using a FACS Aria^TM^ III (BD Biosciences) and the readouts analyzed using the software FlowLogic^TM^ 6 (Inivai Technologies). Gating strategy used in flow cytometry analysis is shown in Supplementary Figure 1.

### Immunohistochemistry

Serial sections were stained for myelin oligodendrocyte glycoprotein (MOG) and hematoxylin and eosin (H&E). Endogenous peroxidases were exhausted by incubation with 0.2% hydrogen peroxide solution (Sigma-Aldrich) in methanol. Slides were then washed in PBS containing 0.2% Triton X-100 (Merck) (PBST) and non-specific staining blocked with 3% bovine serum albumin (BSA) (Sigma-Aldrich). Slides were incubated for 1 h at RT with biotinylated monoclonal mouse anti-MOG (purified from hybridoma cells, provided by Professor Christopher Linington, Glasgow). Sections were then washed in PBST and incubated for 1 h with streptavidin-horseradish peroxidase (GE Healthcare). Slides were developed by 3.3′-Diaminobenzidine (DAB) (SigmaAldrich). For hematoxylin and eosin staining the slides were incubated in ice-cold methanol (Sigma-Aldrich) and then rinsed in distilled water. Subsequently the sections were immersed in Mayer’s hematoxylin staining solution for 15 min (Sigma Aldrich), washed in running water and thereafter stained in 0.5% eosin for 15 s (Merck). Images were acquired using an Olympus BX51 microscope (Olympus) with an Olympus DP73 camera and analyzed using ImageJ software.

### Immunofluorescence Staining

Slides were dried and washed in PBST followed by blocking in 3% BSA. For CD45 immunostaining, tissue sections were incubated with phycoerythrin conjugated anti-CD45 (30-F11, Biolegend) for 1 h at RT. Slides were then incubated for 10 min at RT with DAPI solution (Invitrogen) to visualize nuclei. CD4^+^ T lymphocytes were stained with rat anti-CD4 (191.1.2, hybridoma cell line, provided by Professor Steve Cobbold, Oxford) and Alexa Fluor 558 goat anti-rat IgG (Life Technologies). Axonal damage was investigated using polyclonal rabbit antibody against amyloid precursor protein (APP) (Abcam) and goat anti-rabbit Alexa Fluor 555 (Invitrogen). Subcapsular sinus macrophages in the dcLNs were stained with rabbit anti mouse CD169 (MOMA1, Wako Pure Chemical Industries), and Alexa Fluor 555 donkey anti-rabbit IgG (Life Technologies). Immunofluorescent staining was visualized with an Olympus BX63 microscope (Olympus) and X-cite 120LED (Lumen Dynamics) using Olympus CellSens software. The sections were photographed with an Olympus DP80 digital camera, and the images were processed using ImageJ.

### Quantification of Pathology

MOG staining was analyzed on serial sections spanning the focal lesion in the brain. Demyelination was quantified as the area of loss of MOG staining as a percentage of the area of CC for each section analyzed. The section with the greatest loss from each mouse was chosen as representative for the lesion and used for illustration and statistical analysis. Infiltration identified by hematoxylin and eosin staining was quantified in the same manner, the section showing greatest infiltration was chosen and the area of infiltration was measured and divided by the area of CC.

### Statistical Analysis

Correlation data was first tested for variance between the different focal EAE experiments using Kruskal-Wallis test followed by Dunn’s multiple comparison. All *p*-values were >0.23 indicating no significant difference between the experiments. The experiments were therefore pooled together, and the correlation analyzed using nonparametric Spearman correlation. Data were analyzed via nonparametric Mann Whitney U-test using Graphpad Prism 8 (Graphpad Software Inc). The results are presented as mean ± standard error of mean (SEM) and *P*-values <0.05 were considered statistically significant.

## Results

### Focal EAE Model

Focal brain lesions were induced in C57BL6/J mice at day 10 post immunization for EAE, by stereotactic needle insertion to CC (Figure 1A). Lesions in CC did not occur without needle insertion. However, needle insertion to CC directs lesion pathology which consists of demyelination, detected by loss of MOG staining (Figure 1C), co-localized with cellular infiltrates, detected by H&E staining (Figure 1D). Moreover, APP accumulation localized in the lesion was observed, indicative of axonal damage (Figure 1E; Haines et al., 2011). Similar lesions were induced by intracortical needle insertion (data not shown) but were not included in this study. Initial studies at day 14, 16, and 18 post immunization identified day 16 as the optimal timepoint for CC lesion analysis. All mice that received a stereotactic needle insertion developed focal lesions, including those that were asymptomatic – the percentage showing motor symptoms varied between experiments. Our study was not designed to evaluate EAE grade (motor symptoms) and we did not observe significant amelioration of EAE after MIS416 treatment (Figure 1F). While CC lesions do not lead to motor symptoms, we observed a slight correlation between EAE grade and lesion size (*n* = 23, *r* = 0.632, *p* = 0.0012) (Figure 1G). We interpret this to reflect variability in immunization which would affect both outcomes, although this was not pursued.

### Intrathecal Administration of MIS416 Leads to Reduced Focal Lesion Pathology

To investigate the effect of innate signaling on focal demyelination, focal lesions were induced as described above and on day 12 MIS416 or vehicle was administered via i.t. injection. Brains were collected on day 16 post immunization and sections spanning the focal lesion were stained with anti-MOG to evaluate myelin loss, and with H&E to evaluate leukocyte infiltration. Intrathecal administration of MIS416 significantly reduced myelin loss in the CC when compared to vehicle controls (Figures 1C,H). Infiltration was quantified in the same manner as demyelination and was significantly reduced at the lesion in MIS416-treated mice (Figures 1D,I). Furthermore, we observed decreased staining for APP in the CC after MIS416 treatment (Figure 1E). In the current experiment we did not observe long-term amelioration of EAE after MIS416 treatment (Figure 1F). However, 24 h post i.t. treatment with MIS416 we observed suppression of symptoms, though not significant. Immunofluorescence analysis on day 16 showed increased infiltration in the meninges, identified by CD45 staining, in the MIS416-treated mice compared to control mice, as expected (Figure 1J).

**FIGURE 2:**
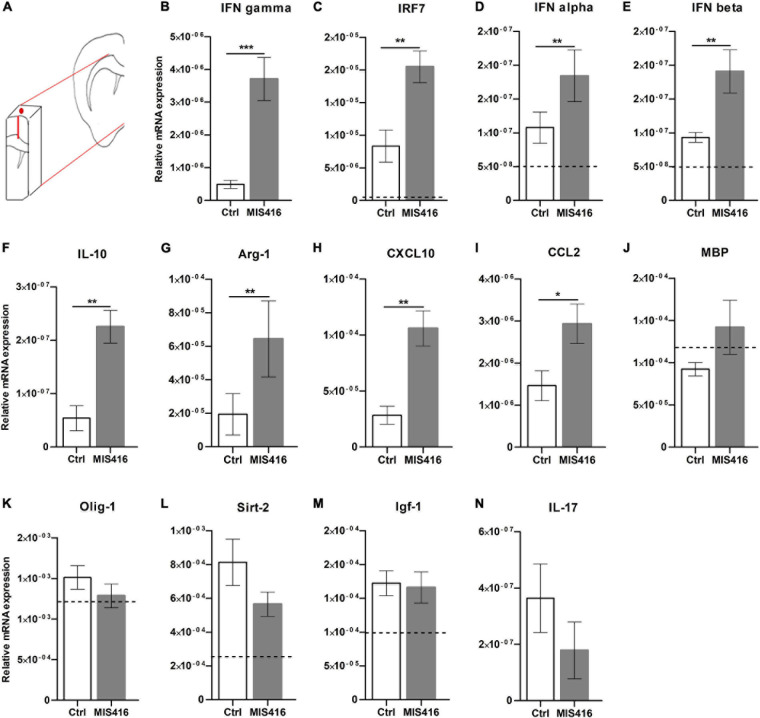
MIS416 affects gene expression at the lesion site. **(A)** A Schematic illustration demonstrating the selected fragment of brain tissue surrounding the lesion site (approx. 50 mg.) which was isolated for RT-qPCR analysis. **(B–N)** Relative expression of the indicated genes at the lesion site (day 16) from MIS416 (*n* = 8) and vehicle (Ctrl, *n* = 7) treated mice with focal EAE. The dotted line represents average levels of genes that were detected in unmanipulated mice (*n* = 5). Data are presented as mean ± SEM. ND: Not detected. Results were analyzed using the two-tailed Mann-Whitney *u*-test; *p < 0.05, **p < 0.01, ***p < 0.001.

To further investigate the mode of action of MIS416, RNA was isolated from microdissected focal lesions (Figure 2A) for RT-qPCR analysis of cytokines and chemokines previously implicated in MIS416-mediated ameliorating effect (White et al., 2018; Khorooshi et al., 2020). The type II interferon IFN-γ (Figure 2B), as well as IFN regulatory factor (IRF) 7 (Figure 2C) and type I IFNs IFN-α (2+6+12+14) and IFN-β (Figures 2D,E), were significantly upregulated after MIS416-treatment. MIS416 also induced the anti-inflammatory cytokine IL-10 (Figure 2F) as well as the enzyme Arg-1 (Figure 2G) that is implicated in tissue repair. Likewise, levels of mRNA for CXCL10 and CCL2 (Figures 2H,I), involved in monocyte and lymphocyte recruitment, were significantly increased in MIS416-treated mice. Level of expression of genes associated with remyelination and oligodendrocyte proliferation such as MBP, Olig-1, Sirt-2, IGF-1 (Figures 2J–M) as well as IL-17 were unaffected by MIS416 treatment (Figure 2N).

### Intrathecal Administration of MIS416 Leads to Increased IL-10 and IFN-γ Expression in Draining Cervical Lymph Nodes

**FIGURE 3:**
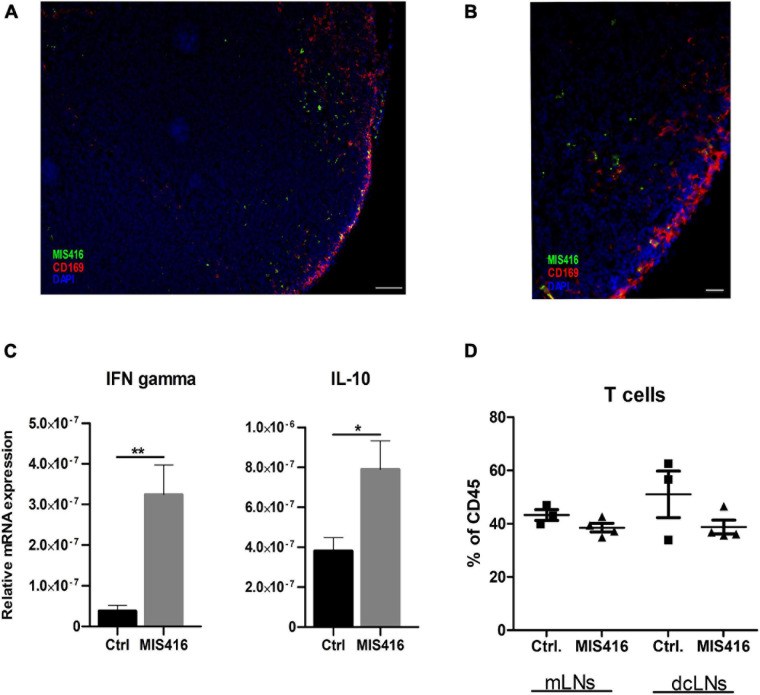
Intrathecally administered fluorescent MIS416 can be detected in cervical draining lymph nodes where it promotes expression of IL-10 and IFN-γ. **(A)** Representative micrographs of mandibular LN from mice with focal EAE treated with intrathecal MIS146 showing colocalization of MIS416 (green) and nodal macrophages expressing CD169 (red); nuclei were stained with DAPI (blue). **(B)** Among cells positive for MIS416 (green) were nodal macrophages expressing CD169 (red). Nuclei were stained with DAPI (blue). Scale bar: A = 100 μm, B = 20 μm. **(C)** RT-qPCR analysis of IFN-γ and IL-10 expression in pooled mandibular and deep cervical LN collected 4 days post intrathecal injections of vehicle (Ctrl, *n* = 5) or MIS416 (*n* = 7). **(D)** FC analysis on mandibular and deep cervical LN (day16) of vehicle (Ctrl, *n* = 3) and MIS416 treated mice (*n* = 3). Data are presented as mean ± SEM. Results were analyzed using the two-tailed Mann-Whitney *u*-test; *p < 0.05, **p < 0.01.

We then investigated whether and how MIS416 effects extended outside the CNS. A major CSF outflow pathway is via the meningeal lymphatic system to the cervical lymph nodes, which include deep cervical lymph nodes (dcLNs) and mandibular LNs (Raper et al., 2016; Ma et al., 2017). Intrathecally-injected MIS416 was detected in both mandibular and dcLNs at day 16 post immunization. The microparticles were located throughout the LNs, but mainly in cortex and cortical sinuses (Figure 3A). MIS416 colocalized with CD169^+^ cells, indicating that subcapsular sinus macrophages were among the cells phagocytosing MIS416 (Figure 3B). Furthermore, gene expression analysis revealed significant upregulation of IFN-γ and IL-10 in the dcLNs (Figure 3C). Flow cytometry analysis showed that the percentage of TCRβ^+^ T-cells in the dcLNs was unaffected by intrathecal MIS416 administration (Figure 3D).

### MIS416 Alters the Composition of CNS Infiltrates

**FIGURE 4:**
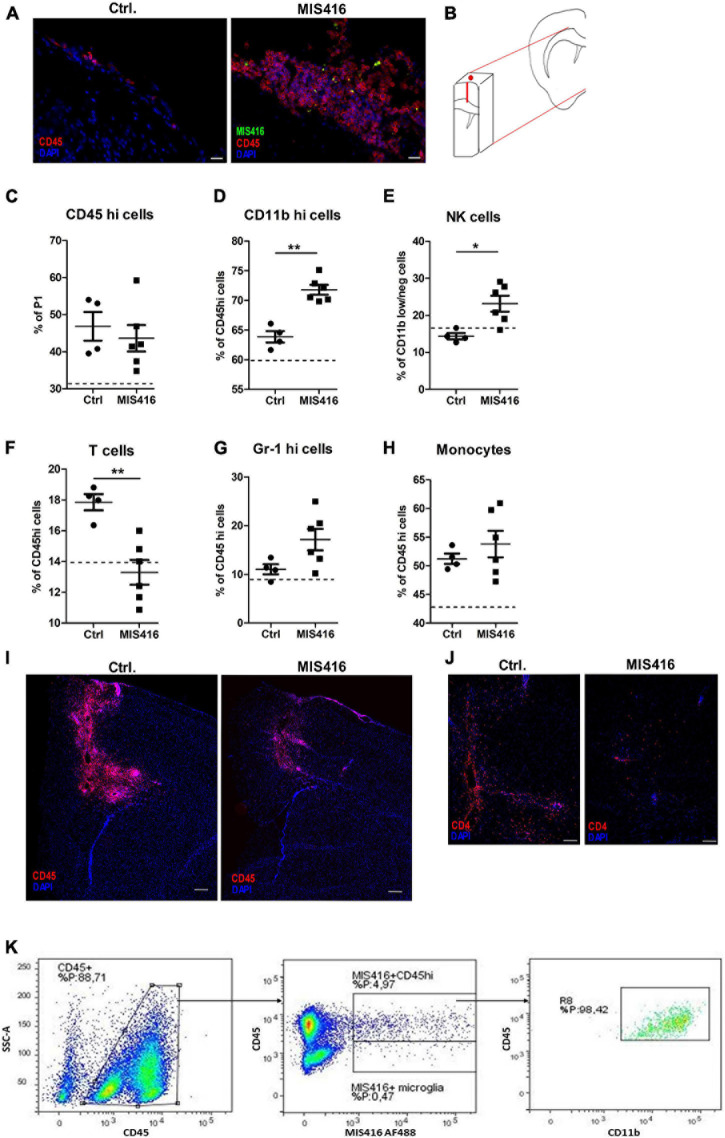
MIS416 treatment influenced cellular infiltrates to the lesion site. **(A)** Representative images showing infiltrating leukocytes in meninges in Ctrl vehicle-treated and MIS416-treated mice (CD45 – red, DAPI – blue, MIS416 – green). **(B)** Schematic illustration demonstrating the piece of brain tissue isolated for FC (50 mg). **(C–H)** Results of FC analysis of brain tissue isolated 24 h post intrathecal injection in focal EAE mice treated with vehicle (Ctrl, *n* = 4) and MIS416 (MIS416, *n* = 6). Shown are percentages for individual mice of CD45^hi^ cells **(C)**, CD11b^hi^ cells **(D)**, NK cells **(E)**, T cells **(F)**, neutrophils **(G)** and monocytes **(H)**. The dotted line represents average percentage levels in unmanipulated mice (*n* = 4). Data are presented as mean ± SEM. Results were analyzed using the two-tailed Mann-Whitney *u*-test; *p < 0.05, **p < 0.01. **(I)** Representative images of the lesion site 24 h post intrathecal injection with vehicle (Ctrl) or MIS416 demonstrating infiltrating leukocytes stained with anti-CD45 (red); cell nuclei were stained with DAPI (blue). **(J)** Representative images of the lesion site 24 h post intrathecal injection with vehicle (Ctrl) or MIS416 demonstrating infiltrating T-cells stained with anti-CD4 (red); cell nuclei were stained with DAPI (blue). **(K)** Representative FC dot-plots showing infiltrating CD45^hi^ cells as well as CD45^dim^ microglia that were positive for MIS416 fluorescence. MIS416 fluorescent CD45hi cells were shown to be CD11b^hi^ myeloid cells. Scale bars: A = 20 μm, D = 200 μm, E = 100 μm.

Immunofluorescence staining revealed that MIS416 was taken up by CD45^+^ cells in the meningeal compartment at 24 h post i.t. injection (Figure 4A). Therefore, a tissue block containing meninges, needle track and the lesion (Figure 4B) was analyzed by flow cytometry to identify changes in the cell composition of CNS infiltrates at this timepoint, to help understand the mechanism behind the reduced pathology at day 16. Cells infiltrating from blood were distinguished from CNS-resident microglia by relative levels of CD45 expression (Prinz et al., 2017). Percentages of CD45^high^ cells in these tissue blocks did not differ between the MIS416 and vehicle control group (Figure 4C), immunofluorescence staining at the same timepoint showed reduced CD45 infiltration at the lesion site itself (Figure 4I).

Characterization of specific lineages within the CD45^high^ population revealed increased percentages of CD11b^high^ (Figure 4D) and natural killer (NK) cells (CD11b^dim^TCRβ^*neg*^NK1.1^+^, Figure 4E) after intrathecal injection of MIS416. Moreover, mice that received intrathecal MIS416 showed significantly reduced percentages of TCRβ^+^ T-cells (Figure 4F). This reduction in T-cells was confirmed by immunofluorescence staining showing reduction in CD4^+^ cells at the lesion site in MIS416-treated compared to the vehicle control group (Figure 4J). There were no significant differences in the percentages of neutrophils (CD45^high^CD11b^high^Gr-1^+^) and monocytes (CD45^high^ CD11b^high^Gr-1^+^ F4/80^+^) between MIS416-treated and vehicle control mice (Figures 4G,H).

Further analysis revealed that both infiltrating CD45^high^ cells and CD45^dim^ microglia had phagocytosed fluorescent-tagged MIS416. The great majority of the CD45^high^ cell population that contained MIS416 particles co-expressed high levels of CD11b which indicated their myeloid origin (Figure 4K).

## Discussion

In this study, we investigated the effect of innate signaling in the CNS on demyelinating lesion pathology in a focal EAE mouse model. Intrathecal administration of the immunomodulatory microparticle MIS416 significantly decreased myelin loss and cellular trafficking into the CC as well as reduced axonal damage. Furthermore, the reduction in demyelinating pathology was associated with increased expression of signature cytokine and chemokine genes involved in MIS416 signaling at the lesion site. DcLNs showed upregulation of IFN-γ and IL-10. Characterization of the CNS infiltrates 24 h post MIS416 administration revealed increased percentages of CD11b^high^ and NK-cells as well as reduction in T-cells.

Here, we show for the first time by employing a focal EAE model, that innate signaling within the CNS reduces myelin loss and prevents the formation of focal brain lesions. MIS416 treatment affected the composition of the CNS infiltrates at the lesion site. We observed an increase in the CD11b^high^ population and NK cells following MIS416 treatment. Importantly, there was a significant reduction in T-cells. This reduction was confirmed by immunofluorescence staining showing decreased infiltration and CD4^+^ T cells in the needle track and CC. This is supported by studies by us and by White and colleagues who demonstrated that MIS416-mediated protection in EAE included expansion of myeloid cells with regulatory properties that were selectively recruited to the CNS (White et al., 2018; Khorooshi et al., 2020). These cells were suggested to suppress and modulate T-cell activity that resulted in decreased trafficking of pathogenic lymphocytes to the CNS (White et al., 2018). Those studies used higher doses (White et al., 2018; Khorooshi et al., 2020), different treatment kinetics (Khorooshi et al., 2020) and injection routes (White et al., 2018). The targeted lesions in this study do not induce motor symptoms.

Previous studies have demonstrated that MIS416-mediated protection is dependent upon type I and II IFN signaling (White et al., 2018; Khorooshi et al., 2020). Consistent with this, our experiments show that intrathecal MIS416 significantly upregulated IFN-γ expression as well as Type I IFNs, IFN-α and IFN-β, at the lesion site. IFN-γ has been regarded primarily as a pro-inflammatory cytokine under various inflammatory conditions and implicated in autoimmune pathologies (Lees and Cross, 2007). On the other hand, IFN-γ has been shown to play contradictory roles and may act as an immune modulator upstream of inflammatory as well as regulatory pathways (Zhang, 2007; Savarin et al., 2012; Arellano et al., 2015). Specifically, White et al. showed that disease-modifying effect of MIS416 in EAE was dependent on innate IFN-γ (White et al., 2018). Type I IFNs play an important role in regulation of CNS inflammation and mediation of anti-inflammatory responses. IFNβ is a therapeutic immunomodulatory agent used in the treatment of relapsing-remitting MS (Bermel and Rudick, 2007). Sustained low doses of IFNα have been shown to improve EAE symptoms and to modulate immune responses by inducing Tregs and reducing inflammatory infiltrates (Vasquez et al., 2019). Furthermore, it has been shown that Type I Interferons can drive the production of IFN-γ by NK-cells (Hunter et al., 1997; Thio et al., 2019), which in turn might be responsible for the increased IFN-γ message observed in MIS416-treated mice. Additionally, studies have identified a regulatory role for NK cells in EAE, showing that NK cells can directly kill encephalitogenic T cells, and thus ameliorate EAE (Zhang et al., 1997; Xu et al., 2005; Huang et al., 2006; Boros et al., 2016). This may contribute to the observed reduction in T-cells at the lesion site. Furthermore, a recent study by Sanmarco et al. identified IFN-γ produced by meningeal NK-cells as the driver of anti-inflammatory responses in astrocytes that can dampen CNS inflammation (Sanmarco et al., 2021). Taken together, simultaneous stimulation of TLR9 and NOD2 receptors appears to induce selective infiltration to the lesion site and reduce lesion formation. Although the mechanistic basis for this selectivity remains unknown, other innate ligands such as poly-I:C (ligand for TLR3) have been shown to recruit myeloid cells into the CNS and attenuate EAE, and ligands for TLR7 and TLR9 have also been shown to reduce EAE disease severity (Hayashi et al., 2012; Longhini et al., 2014; Owens et al., 2020).

Accumulating evidence suggests that stimulation of innate receptors in myeloid cells may induce a phenotypic switch to induce cells with regulatory properties (Boros et al., 2016; White et al., 2018; Khorooshi et al., 2020). In the context of cancer, it is well accepted that suppressive myeloid cells can inhibit T-cell responses by producing the enzyme Arg-1 (Rath et al., 2014; Steggerda et al., 2017). Our experiments showed significant upregulation of Arg-1 at the lesion site. In the CNS, Arg-1 has also been suggested to be primarily expressed by infiltrating myeloid cells and increased expression corresponds with recovery from injury or disease (Greenhalgh et al., 2016). Furthermore, it has been shown that activated neutrophils following ischemic stroke are a major source of Arg-1 that leads to T-cell dysfunction and immunosuppression (Sippel et al., 2015). In a previous study we showed that intrathecal MIS416 induced infiltration of suppressive neutrophils which could transfer protection against EAE (Khorooshi et al., 2020). In the present work we did not find differences in the percentages of neutrophils at the lesion site. Due to lack of markers to assess whether MIS416 treatment prompted infiltration of neutrophils with suppressive properties we did not examine this further.

In contrast to the brain, we did not observe significant changes in the proportion of T-cells in the dcLNs. Increasing the number of animals in each group could help to substantiate the current findings. However, there was significant upregulation of two cytokines associated with MIS416-mediated effect, IFN-γ and IL-10. White et al. demonstrated that MIS416-mediated suppression of CD4 T cell proliferation is dependent on alterations in the immune environment of the lymphoid tissue (White et al., 2018). Although systemic disease suppression was not a feature of our model, it remains to be determined whether altered immune responses in dcLNs could have contributed to reduced focal brain pathology.

In conclusion, we have demonstrated that stimulation of innate receptors in the CNS prevents the formation of focal lesions in the brain. The focal EAE model that we describe represents a useful experimental system to study the mechanisms of demyelination as well as to investigate potential therapeutic agents that can influence lesion development. Our observations suggest that intrathecal treatment with selected innate ligands alters the composition of cellular infiltrates at the lesion site as well as showing effect on CNS-draining LN, which together result in decreased focal EAE pathology.

## Data Availability Statement

The original contributions presented in the study are included in the article/Supplementary Material, further inquiries can be directed to the corresponding author/s.

## Ethics Statement

The animal study was reviewed and approved by Animal Experiments inspectorate under Danish Ministry of Food, Agriculture and Fisheries, The Danish Veterinary and Food Administration, approval identification number: 2020-15-0201-00652.

## Author Contributions

MD and JM wrote the original manuscript, performed the experiments, statistical analysis, and were involved in study design and development. RK and MD performed the stereotactic and intrathecal injections. TO and RK gave supervision on the design of the study and helped to draft the manuscript. MM provided additional data on the focal EAE model. GW provided and advised on use of MIS416. TO, RK, MM, MD, AW, and KJ were involved in the establishment of the focal EAE model. All authors contributed to revision and approved the final version of the manuscript.

## Conflict of Interest

GW was employed by the company Innate Immunotherapeutics. The remaining authors declare that the research was conducted in the absence of any commercial or financial relationships that could be construed as a potential conflict of interest.

## Abbreviations

Arg-1: arginase-1
CC: corpus callosum
CCL2: monocyte chemoattractant protein-1
CNS: central nervous system
CSF: cerebrospinal fluid
CXCL-10: C-X -C motif chemokine 10
EAE: experimental autoimmune encephalomyelitis
IFN: interferon
IL: interleukin
IRF: interferon regulatory factor
i.t.: intrathecal
LNs: lymph nodes
dcLN: deep cervical lymph nodes
mLN, mandibular lymph nodes MS: multiple sclerosis
MOG: myelin oligodendrocyte glycoprotein
NK-cells: natural killer cells
NOD: nucleotide-binding oligomerization domain
TLR: toll-like receptor.
