# Supplementary material for: Innate Signaling in the CNS Prevents Demyelination in a Focal EAE Model
Source: Front Neurosci. 2021 Jun 3;15:682451. doi: 10.3389/fnins.2021.682451 (PMC8209300; doi:10.3389/fnins.2021.682451)
Supplement: Supplementary file 1 [file Data_Sheet_1.PDF]

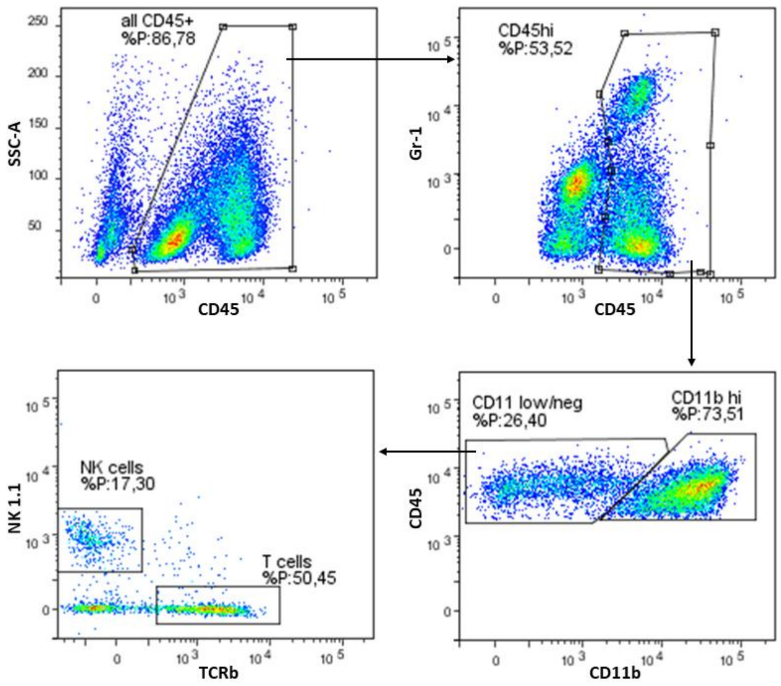

Supplementary Fig. 1. Representative images demonstrating gating strategy used for FC analysis from the collected brain tissue. After excluding debris and doublets all immune cells were identified based on CD45 expression. Next, on the basis of CD45 expression, cells were gated as CD45^{dim} microglia and CD45^{hi} infiltrating cells. CD45^{hi} cells were further divided into myeloid populations characterized by high expression of CD11b and cells of lymphoid lineage without or low expression of CD11b. Among CD11b$^{low/neg}$ population T cells were distinguished based on TCRβ expression and NK cells gated on NK1.1 expression and the absence of TCRβ.
